# Supplementary material for: Tau deposition patterns are associated with functional connectivity in primary tauopathies
Source: Nat Commun. 2022 Mar 15;13:1362. doi: 10.1038/s41467-022-28896-3 (PMC8924216; doi:10.1038/s41467-022-28896-3)
Supplement: Supplementary file 1 — Supplementary Information [file 41467_2022_28896_MOESM1_ESM.pdf]

Tau deposition patterns are associated with functional connectivity in primary tauopathies

Supplementary information

Supplementary Figure 1:

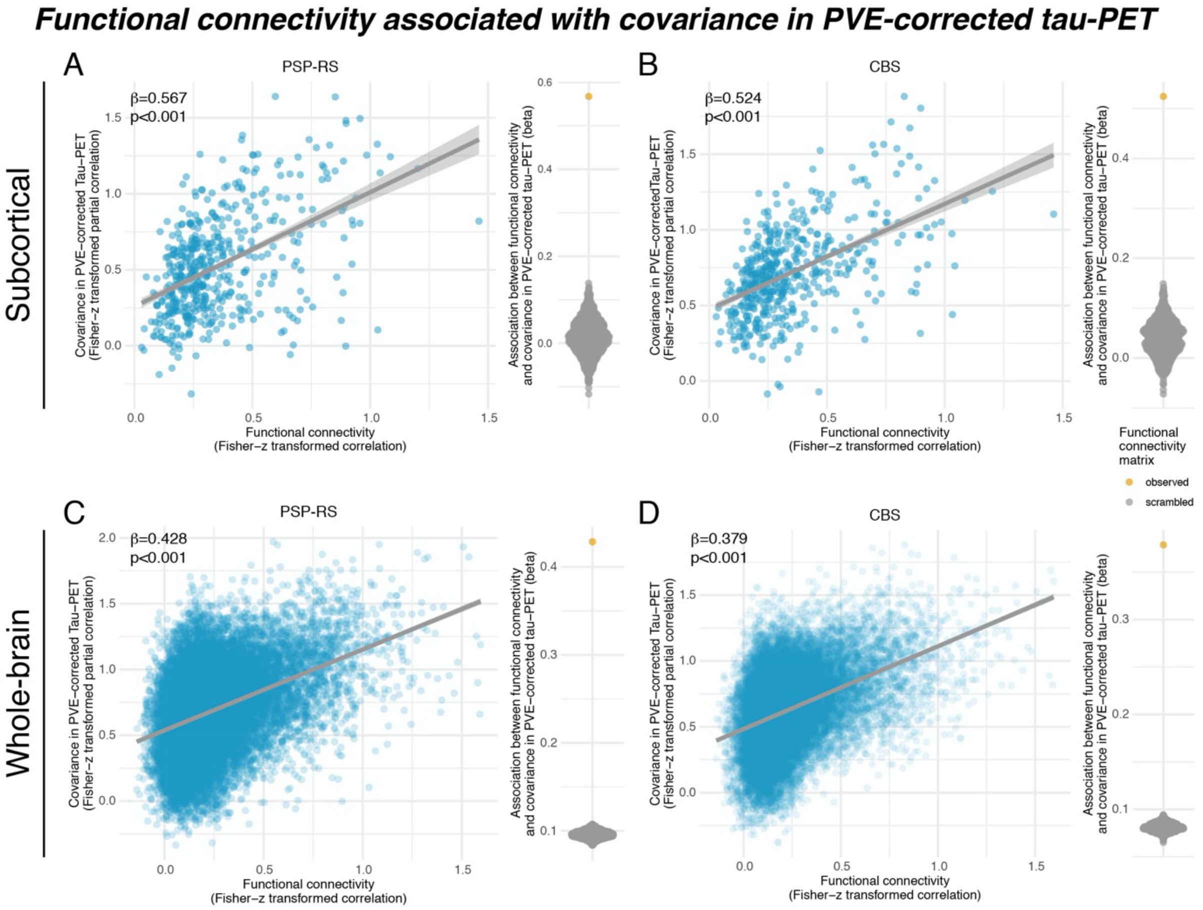

Scatterplots illustrating the association between functional connectivity and covariance in PVE-corrected 18-F-PI2620-PET among subcortical regions in PSP-RS (A) and CBS groups (B), as well as among subcortical and cortical regions for PSP-RS (C) and CBS groups (D). Standardized beta- and p-values were derived from linear regression controlling for Euclidean distance between ROIs. Beeswarm plots illustrate the distribution of standardized beta-values derived from repeating the analysis 1000 times using scrambled connectomes with preserved weight- and degree-distribution (grey points) vs. the beta value derived from the association with the actual observed connectivity matrix that is illustrated in the scatterplot (yellow point). Two-sided p-values have been determined via linear regression. Linear model fits are indicated together with 95% confidence intervals. Source data are provided as a Source Data file.

Supplementary Figure 2:

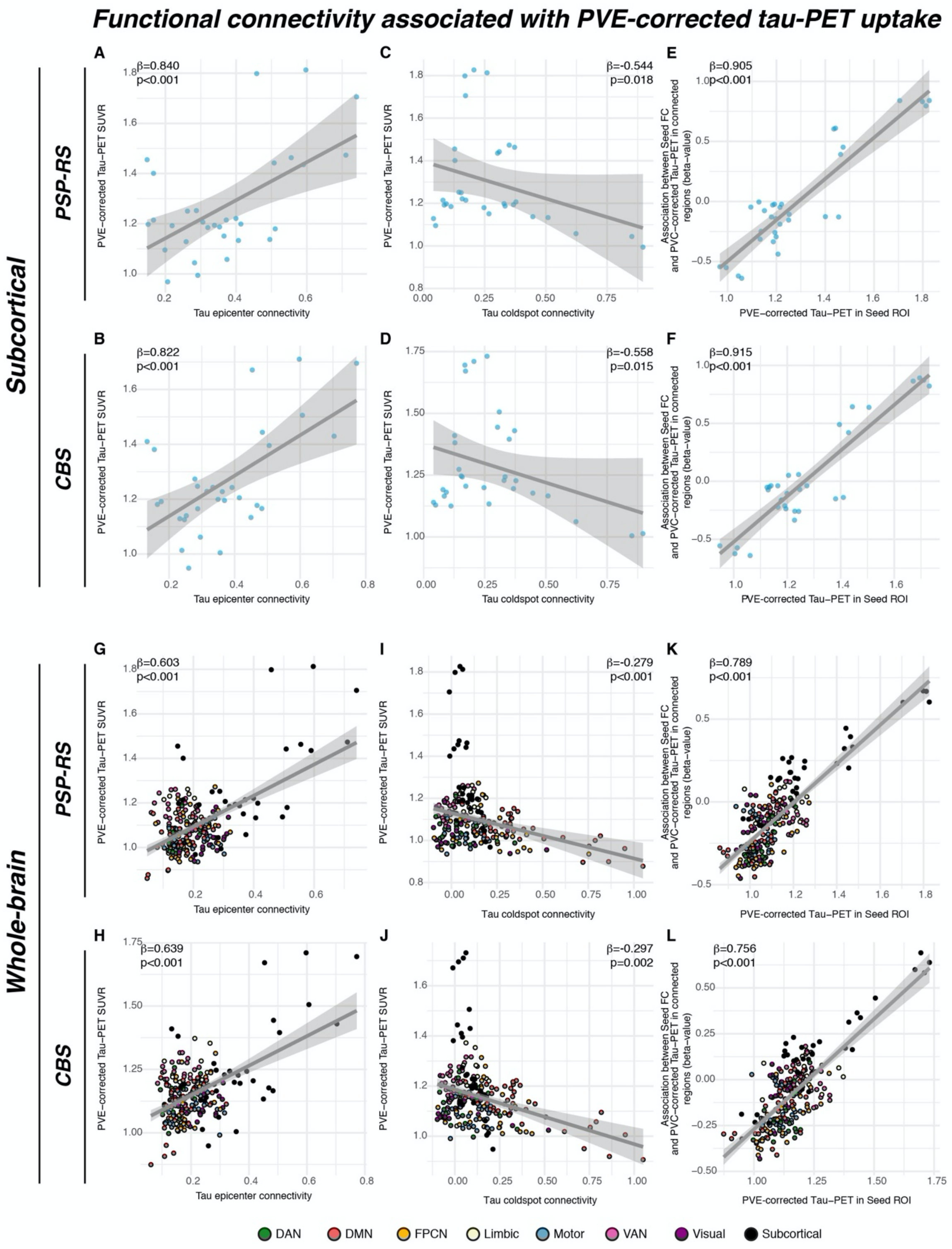

Associations between group-average subcortical PVE-corrected 18-F-Pi2620-PET data and seed-based functional connectivity of tau epicenters (i.e. regions with highest group-average tau) in PSP-RS (A) and CBS (B), illustrating that regions with high connectivity to the tau epicenter show high tau-PET. The same association was plotted for tau coldspots (i.e. regions with lowest tau-PET) for PSP-RS (C) and CBS (D), illustrating that regions closely connected to the tau coldspots show also low tau-PET. Standardized beta- and p-values were derived from linear regression controlling for Euclidean distance between ROIs. The analysis was repeated for all ROIs, and the respective seed ROIs tau-PET uptake was plotted against the regression derived beta-value, showing that seed regions with higher tau-PET show a positive association between seed-based connectivity and tau-PET in connected regions, whereas regions with lower tau-PET show a negative association between seed-based connectivity and tau-PET in connected regions in PSP (E) and CBS (F). These findings indicate that seed

ROIs are preferentially connected to other regions with similar tau-PET levels. All analyses were repeated including using the combined set of 200 cortical and 32 subcortical ROIs, showing a fully consistent result pattern across the entire brain (G-L). Two-sided p-values have been determined via linear regression. Linear model fits are indicated together with 95% confidence intervals. Source data are provided as a Source Data file.

Supplementary Figure 3:

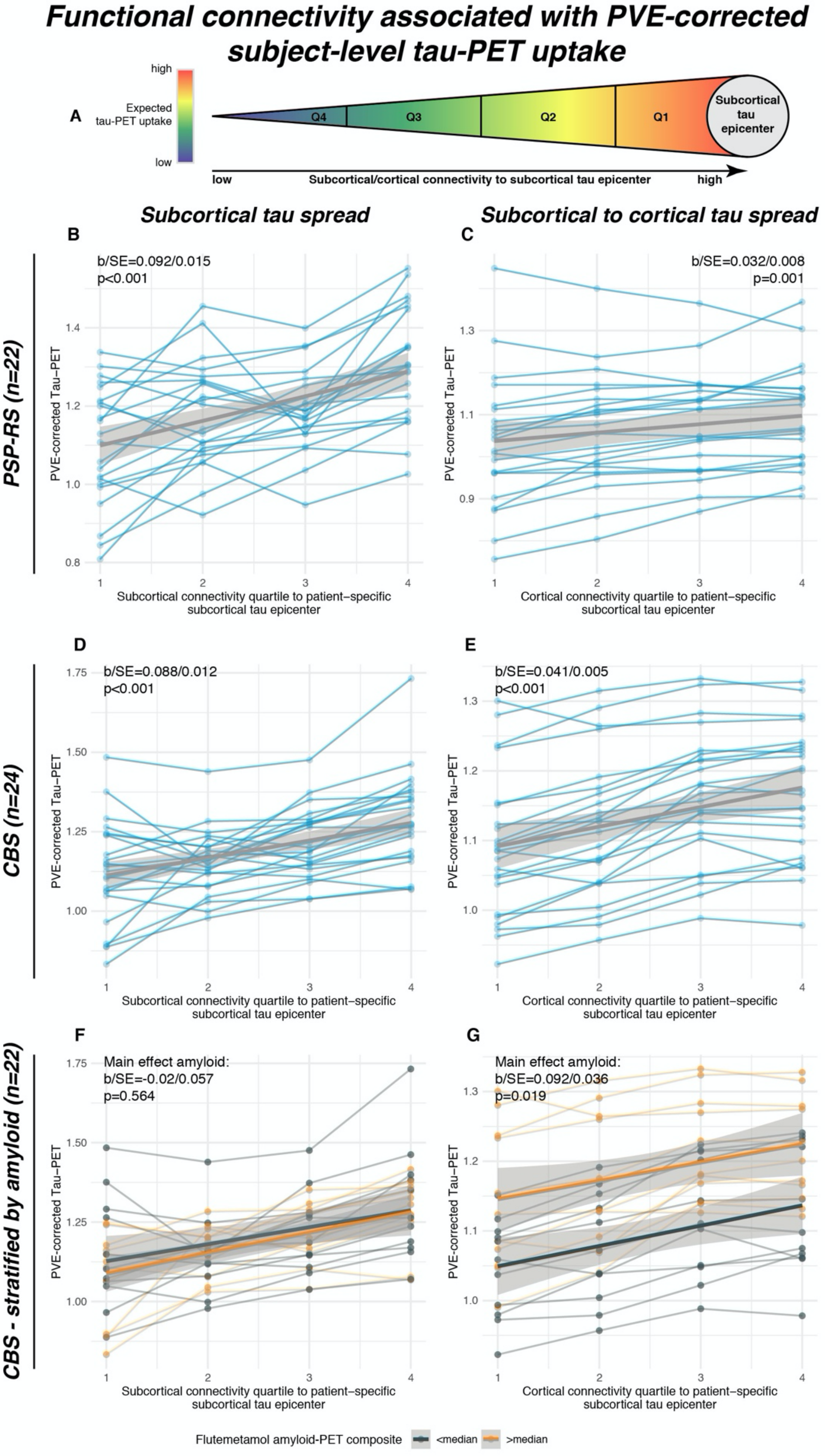

Using subject-level PVE-corrected tau-PET data, we determined for each PSP-RS and CBS patient the subcortical tau epicenter (A), i.e. defined as 20% of ROIs with highest tau-PET SUVRs. The remaining ROIs were grouped for each subject into quartiles, depending on connectivity strength to the subject-specific tau epicenter. Highest tau-PET was expected for regions most closely connected to the tau epicenter (i.e. Q1) whereas lowest tau-PET was expected for ROIs only weakly connected to the tau epicenter. Subject-specific tau-

PET data for subcortical Q1-Q4 ROIs (B&D) as well cortical Q1-Q4 ROIs (C&E) is shown, illustrating that tau-PET was highest in subcortical and cortical regions that are most closely connected to the subcortical tau epicenter (i.e. Q1), with gradual decreases across less strongly connected regions. For a subset of CBS patients (n=22), we further stratified these analyses by above vs. below median global amyloid-PET SUVRs (i.e. subthreshold amyloid, as all subjects were amyloid negative on visual read). Amyloid-stratified analyses illustrate that above median amyloid levels were not associated with elevated tau spread from subcortical epicenters to subcortical Q1-Q4 ROIs (F), but with increased tau spread from subcortical epicenters to cortical Q1-Q4 ROIs (G). All statistical indices (i.e. b-values, standard errors and p-values) were derived from linear mixed models, controlling for age, sex, study center, mean Euclidean distance of Q1-Q4 ROIs to the tau epicenter and random intercept. Two-sided p-values have been determined via linear mixed effects models. Linear model fits are indicated together with 95% confidence intervals. Source data are provided as a Source Data file.

Supplementary Note: RIDs of ADNI resting-state fMRI participants

0021, 0031, 0059, 0069, 0127, 0156, 0337, 0413, 0677, 0969, 1169, 1261, 4037, 4043, 4076, 4084, 4105, 4148, 4164, 4200, 4213, 4224, 4313, 4365, 4367, 4367, 4369, 4376, 4384, 4387, 4399, 4399, 4410, 4427, 4446, 4448, 4453, 4469, 4483, 4488, 4488, 4580, 4585, 4598, 4643, 4644, 4649, 4785, 4856, 5078, 5079, 5093, 5100, 5140, 5169, 5175, 5178, 5193, 5194, 5200, 5203, 5214, 5242, 5243, 5253, 5266, 5266, 5288, 0120

Supplementary Table 1: Group-level analyses, non-PVE-corrected

			PSP-RS					CBS			
			controlling for Euclidean distance		no control for Euclidean distance			controlling for Euclidean distance		no control for Euclidean distance	
Analysis	***Atlas***	***Figure***	***β***	***P***	***β***	***p***	***Figure***	***β***	***p***	***β***	***p***
**Functional connectivity vs. Covariance in tau-PET**	**Subcortical**	**3A**	0.616	<0.001	0.532	<0.001	**3B**	0.561	<0.001	0.555	<0.001
	**Subcortical & Cortical**	**3C**	0.450	<0.001	0.410	<0.001	**3D**	0.402	<0.001	0.429	<0.001
**Tau epicenter connectivity vs. Tau-PET SUVR**	**Subcortical**	**4A**	0.880	<0.001	0.733	<0.001	**4B**	0.933	<0.001	0.781	<0.001
	**Subcortical & Cortical**	**4G**	0.688	<0.001	0.659	<0.001	**4H**	0.689	<0.001	0.634	<0.001
**Tau coldspot connectivity vs. Tau-PET SUVR**	**Subcortical**	**4C**	-0.613	0.007	-0.375	0.038	**4D**	-0.617	0.006	-0.359	0.047
	**Subcortical & Cortical**	**4I**	-0.241	0.002	-0.283	<0.001	**4J**	-0.242	0.002	-0.306	<0.001
**Tau-PET in seed ROI vs. Association between Seed FC and Tau-PET in connected regions (beta value)**	**Subcortical**	**4E**	0.929	<0.001	0.910	<0.001	**4F**	0.937	<0.001	0.930	<0.001
	**Subcortical & Cortical**	**4K**	0.788	<0.001	0.760	<0.001	**4L**	0.761	<0.001	0.740	<0.001

Supplementary Table 2: Subject-level analyses, non-PVE-corrected

		PSP-RS							CBS						
			controlling for Euclidean distance			no control for Euclidean distance				controlling for Euclidean distance			no control for Euclidean distance		
	Atlas	***Figure***	***b***	***SE***	***p***	***b***	***SE***	***p***	***Figure***	***b***	***SE***	***p***	***b***	***SE***	***p***
**Subcoortical connectivty quartile to patient-specific tau epicenter vs. Tau-PET**	**Subcortical**	**5B**	0.097	0.015	<0.001	0.057	0.007	<0.001	**5D**	0.086	0.013	<0.001	0.057	0.006	<0.001
	**Cortical**	**5C**	0.036	0.008	<0.001	0.025	0.003	<0.001	**5E**	0.032	0.005	<0.001	0.032	0.002	<0.001
**Subcoortical connectivty quartile to patient-specific tau epicenter vs. Tau-PET stratified by amyloid**	**Subcortical**								**5F**	-0.02	0.051	0.653	-0.015	0.049	0.759
	**Cortical**								**5G**	0.078	0.006	0.031	0.078	0.006	0.031

Supplementary Table 3: Group-level analyses, PVE-corrected

		PSP-RS					CBS				
			controlling for Euclidean distance		no control for Euclidean distance			controlling for Euclidean distance		no control for Euclidean distance	
Analysis	***Atlas***	***Figure***	β	***p***	β	***p***	***Figure***	β	***p***	β	***p***
**Functional connectivity vs. Covariance in tau-PET**	**Subcortical**	**Supp. Fig 1A**	0.567	<0.001	0.484	<0.001	**Supp. Fig 1B**	0.524	<0.001	0.515	<0.001
	**Subcortical & Cortical**	**Supp. Fig 1C**	0.428	<0.001	0.382	<0.001	**Supp. Fig 1D**	0.379	<0.001	0.402	<0.001
**Tau epicenter connectivity vs. Tau-PET SUVR**	**Subcortical**	**Supp. Fig 2A**	0.84	<0.001	0.567	<0.001	**Supp. Fig 2B**	0.822	<0.001	0.601	<0.001
	**Subcortical & Cortical**	**Supp. Fig 2G**	0.603	<0.001	0.559	<0.001	**Supp. Fig 2H**	0.639	<0.001	0.516	<0.001
**Tau coldspot connectivity vs. Tau-PET SUVR**	**Subcortical**	**Supp. Fig 2C**	-0.544	0.018	-0.321	0.039	**Supp. Fig 2D**	-0.558	0.015	-0.325	0.037
	**Subcortical & Cortical**	**Supp. Fig 2I**	-0.279	<0.001	-0.297	<0.001	**Supp. Fig 2J**	-0.297	<0.001	-0.348	<0.001
**Tau-PET in seed ROI vs. Association between Seed FC and Tau-PET in connected regions (beta value)**	**Subcortical**	**Supp. Fig 2E**	0.91	<0.001	0.89	<0.001	**Supp. Fig 2F**	0.91	<0.001	0.9	<0.001
	**Subcortical & Cortical**	**Supp. Fig 2K**	0.789	<0.001	0.74	<0.001	**Supp. Fig 2L**	0.756	<0.001	0.7	<0.001

Supplementary Table 4: Subject-level analyses, PVE-corrected

		PSP-RS							CBS						
			controlling for Euclidean distance			no control for Euclidean distance				controlling for Euclidean distance			no control for Euclidean distance		
	Atlas	***Figure***	***b***	***SE***	***p***	***b***	***SE***	***p***	***Figure***	***b***	***SE***	***p***	***b***	***SE***	***p***
**Subcoortical connectivty quartile to patient-specific tau epicenter vs. Tau-PET**	**Subcortical**	**Supp. Fig 3B**	0.092	0.015	<0.001	0.063	0.007	<0.001	**Supp. Fig 3D**	0.088	0.012	<0.001	0.055	0.06	<0.001
	**Cortical**	**Supp. Fig 3C**	0.032	0.008	0.001	0.02	0.003	<0.001	**Supp. Fig 3E**	0.041	0.005	<0.001	0.028	0.002	<0.001
**Subcoortical connectivty quartile to patient-specific tau epicenter vs. Tau-PET stratified by amyloid**	**Subcortical**								**Supp. Fig 3F**	-0.02	0.057	0.564	-0.023	0.054	0.667
	**Cortical**								**Supp. Fig 3G**	0.092	0.036	0.019	0.092	0.036	0.0176

Full member list of the German Imaging Initiative for Tauopathies (GII4T)

Johannes Levin1, Jonathan Vöglein1, Urban Fietzek1, Sonja Schönecker1, Georg Nübling1, Catharina Prix1, Kai Bötzel1, Adrian Danek1, Carla Palleis1, Endy Weidinger1, Sabrina Katzdobler1, Matthias Brendel2, Mengmeng Song2, Alexander Nitschmann2, Maike Kern2, Gloria Biechele2, Anika Finze2, Leonie Beyer2, Peter Bartenstein2, Stefanie Harris2, Julia Schmitt2, Florian Eckenweber2, Simon Lindner2, Franz-Joseph Gildehaus2, Emanuel Joseph2, Maximilian Scheifele2, Christian Zach2, Robert Perneczky3, Jan Häckert3, Boris-Stephan Rauchmann4, Sophia Stöcklein4, Günter Höglinger5, Gesine Respondek5, Henryk Barthel6, Marianne Patt6, Andreas Schildan6, Osama Sabri6, Michael Rullmann6, Joseph Classen7, Dorothee Saur7, Jost-Julian Rumpf7, Matthias L. Schroeter8, Matthias Höllerhage9, Alexander Drzezga10, Thilo van Eimeren10, Jochen Hammes10, Bernd Neumaier10, Michael T. Barbe11, Oezguer Onur11, Estrella Morenas-Rodriguez12, Jochen Herms12, Sigrun Roeber12, Thomas Arzberger12, Christian Haass12, Frank Jessen12, Andrew Stephens13, Norman Koglin13, Andre Mueller13

1. LMU Munich, Dept. Neurology
2. LMU Munich, Dept. Nuclear Medicine
3. LMU Munich, Dept. Psychiatry and Psychotherapy
4. LMU Munich, Dept. Radiology
5. Hannover Medical School, Dept of Neurology
6. University of Leipzig, Dept. Nuclear Medicine
7. University of Leipzig, Dept. of Neurology
8. Max-Plank-Institute of Human Cognitive and Brain Sciences Leipzig
9. Technical University of Munich, Dept. Neurology
10. University of Cologne, Dept. Nuclear Medicine and Forschungszentrum Jülich
11. University of Cologne, Dept. Neurology
12. DZNE Munich/Bonn
13. Life Molecular Imaging

Full member list of the Alzheimer's disease neuroimaging initiative (ADNI)

Michael Weiner1, Paul Aisen2, Ronald Petersen3, Clifford R. Jack3, William Jagust4, John Q. Trojanowki5, Arthur W. Toga6, Laurel Beckett7, Robert C. Green8, Andrew J. Saykin9, John Morris10, Leslie M. Shaw11, Enchi Liu12, Tom Montine13, Ronald G. Thomas2, Michael Donohue2, Sarah Walter2, Devon Gessert2, Tamie Sather2, Gus Jiminez2, Danielle Harvey7, Matthew Bernstein3, Nick Fox14, Paul Thompson15, Norbert Schuff1, Charles DeCArli7, Bret Borowski3, Jeff Gunter3, Matt Senjem3, Prashanthi Vemuri3, David Jones3, Kejal Kantarci3, Chad Ward3, Robert A. Koeppe16, Norm Foster17, Eric M. Reiman18, Kewei Chen18, Chet Mathis19, Susan Landau4, Nigel J. Cairns10, Erin Householder10, Lisa Taylor Reinwald10, Virginia Lee20, Magdalena Korecka20, Michal Figurski20, Karen Crawford6, Scott Neu6, Tatiana M. Foroud9, Steven Potkin21, Li Shen9, Faber Kelley9, Sungeun Kim9, Kwangsik Nho9, Zaven Kachaturian22, Richard Frank23, Peter J. Snyder24, Susan Molchan25, Jeffrey Kaye26, Joseph Quinn26, Betty Lind26, Raina Carter26, Sara Dolen26, Lon S. Schneider27, Sonia Pawluczyk27, Mauricio Beccera27, Liberty Teodoro27, Bryan M. Spann27, James Brewer28, Helen Vanderswag28, Adam Fleisher18, 28, Judith L. Heidebrink16, Joanne L. Lord16, Sara S. Mason3, Colleen S. Albers3, David Knopman3, Kris Johnson3, Rachelle S. Doody29, Javier Villanueva Meyer29, Munir Chowdhury29, Susan Rountree29, Mimi Dang29, Yaakov Stern30, Lawrence S. Honig30, Karen L. Bell30, Beau Ances10, John C. Morris10, Maria Carroll10, Sue Leon10, Mark A. Mintun10, Stacy Schneider10, Angela Oliver10, Daniel Marson31, Randall Griffith31, David Clark31, David Geldmacher31, John Brockington31, Erik Roberson31, Hillel Grossman32, Effie Mitsis32, Leyla deToledo-Morrell33, Raj C. Shah33, Ranjan Duara34, Daniel Varon34, Maria T. Greig34, Peggy Roberts34, Marilyn Albert35, Chiadi Onyike35, Daniel D'Agostino II35, Stephanie Kielb35, James E. Galvin36, Dana M. Pogorelec36, Brittany Cerbone36, Christina A. Michel36, Henry Rusinek36, Mony J de Leon36, Lidia Glodzik36, Susan De Santi36, P. Murali Doraiswamy37, Jeffrey R. Petrella37, Terence Z. Wong37, Steven E. Arnold11, Jason H. Karlawish11, David Wolk11, Charles D. Smith38, Greg Jicha38, Peter Hardy38, Partha Sinha38, Elizabeth Oates38, Gary Conrad38, Oscar L. Lopez19, MaryAnn Oakley19, Donna M. Simpson19, Anton P. Porsteinsson39, Bonnie S. Goldstein39, Kim Martin39, Kelly M. Makino39, M. Saleem Ismail39, Connie Brand39, Ruth A. Mulnard40, Gaby Thai40, Catherine Mc Adams Ortiz40, Kyle Womack41, Dana Mathews41, Mary Quiceno41, Ramon Diaz Arrastia41, Richard King41, Myron Weiner41, Kristen Martin Cook41, Michael DeVous41, Allan I. Levey42, James J. Lah42, Janet S. Cellar42, Jeffrey M. Burns43, Heather S. Anderson43, Russell H. Swerdlow43, Liana Apostolova44, Kathleen Tingus44, Ellen Woo44, Daniel H.S. Silverman44, Po H. Lu44, George Bartzokis44, Neill R Graff Radford3, Francine Parfitt3, Tracy Kendall3, Heather Johnson3, Martin R. Farlow9, Ann Marie Hake9, Brandy R. Matthews9, Scott Herring9, Cynthia Hunt9, Christopher H. van Dyck45, Richard E. Carson45, Martha G. MacAvoy45, Howard Chertkow46, Howard Bergman46, Chris Hosein46, Sandra Black47, Bojana Stefanovic47, Curtis Caldwell47, Ging Yuek Robin Hsiung48, Howard Feldman48, Benita Mudge48, Michele Assaly48, Andrew Kertesz49, 72, John Rogers49, 72, Dick Trost49, Charles Bernick50, Donna Munic50, Diana Kerwin51, Marek Marsel Mesulam51, Kristine Lipowski51, Chuang Kuo Wu51, Nancy Johnson51, Carl Sadowsky52, Walter Martinez52, Teresa Villena52, Raymond Scott Turner53, Kathleen Johnson53, Brigid Reynolds53, Reisa A. Sperling8, Keith A. Johnson8, Gad Marshall8, Meghan Frey8, Jerome Yesavage54, Joy L. Taylor54, Barton Lane54, Allyson Rosen54, Jared Tinklenberg54, Marwan N. Sabbagh55, Christine M. Belden55, Sandra A. Jacobson55, Sherye A. Sirrel55, Neil Kowall56, Ronald Killiany56, Andrew E. Budson56, Alexander Norbash56, Patricia Lynn Johnson56, Thomas O. Obisesan57, Saba Wolday57, Joanne Allard57, Alan Lerner58, Paula Ogrocki58, Leon Hudson58, Evan Fletcher59, Owen Carmichael59, John Olichney59, Charles DeCarli59, Smita Kittur60, Michael Borrie61, T Y Lee61, Dr Rob Bartha61, Sterling Johnson62, Sanjay Asthana62, Cynthia M. Carlsson62, Steven G. Potkin40, Adrian Preda40, Dana Nguyen40, Pierre Tariot18, Stephanie Reeder18, Vernice Bates63, Horacio Capote63, Michelle Rainka63, Douglas W. Scharre64, Maria Kataki64, Anahita Adeli64, Earl A. Zimmerman65, Dzintra Celmins65, Alice D. Brown65, Godfrey D. Pearlson66, Karen Blank66, Karen Anderson66, Robert B. Santulli67, Tamar J. Kitzmiller67, Eben S. Schwartz67, Kaycee M. Sink68, Jeff D. Williamson68, Pradeep Garg68, Franklin Watkins68, Brian R. Ott69, Henry Querfurth69, Geoffrey Tremont69, Stephen Salloway70, Paul Malloy70, Stephen Correia70, Howard J. Rosen1, Bruce L. Miller1, Jacobo Mintzer71, Kenneth Spicer71, David Bachman71, Elizabether Finger72, Stephen Pasternak72, Irina Rachinsky72, Dick Drost72, Nunzio Pomara73, Raymundo Hernando73, Antero Sarrael73, Susan K. Schultz74, Laura L. Boles Ponto74, Hyungsub Shim74, Karen Elizabeth Smith74, Norman Relkin75, Gloria Chaing75, Lisa Raudin75, Amanda Smith76, Kristin Fargher76, Balebail Ashok Raj76

1. UC San Francisco
2. UC San Diego
3. Mayo Clinic
4. UC Berkeley
5. U Pennsylvania

6. USC
7. UC Davis
8. Brigham and Women's Hospital
9. Indiana University
10. Washington University St. Louis
11. University of Pennsylvania
12. Janssen Alzheimer Immunotherapy
13. University of Washington
14. University of London
15. USC School of Medicine
16. University of Michigan
17. University of Utah
18. Banner Alzheimer's Institute
19. University of Pittsburgh
20. UPenn School of Medicine
21. UC Irvine
22. Khachaturian Radebaugh & Associates
23. General Electric
24. Brown University
25. National Institute on Aging/National Institutes of Health
26. Oregon Health and Science University
27. University of Southern California
28. University of California San Diego
29. Baylor College of Medicine
30. Columbia University Medical Center
31. University of Alabama Birmingham
32. Mount Sinai School of Medicine
33. Rush University Medical Center
34. Wien Center
35. Johns Hopkins University
36. New York University
37. Duke University Medical Center
38. University of Kentucky
39. University of Rochester Medical Center
40. University of California Irvine
41. University of Texas Southwestern Medical School
42. Emory University
43. University of Kansas Medical Center
44. University of California Los Angeles
45. Yale University School of Medicine
46. McGill Univ. Montreal Jewish General Hospital
47. Sunnybrook Health Sciences Ontario
48. U.B.C. Clinic for AD & Related Disorders
49. Cognitive Neurology St. Joseph's Ontario
50. Cleveland Clinic Lou Ruvo Center for Brain Health
51. Northwestern University
52. Premiere Research Inst Palm Beach Neurology
53. Georgetown University Medical Center
54. Stanford University
55. Banner Sun Health Research Institute
56. Boston University
57. Howard University
58. Case Western Reserve University
59. University of California Davis
60. Neurological Care of CNY
61. Parkwood Hospital
62. University of Wisconsin
63. Dent Neurologic Institute
64. Ohio State University
65. Albany Medical College
66. Hartford Hosp Olin Neuropsychiatry Research Center
67. Dartmouth Hitchcock Medical Center

68. Wake Forest University Health Sciences
69. Rhode Island Hospital
70. Butler Hospital
71. Medical University South Carolina
72. St. Joseph’s Health Care
73. Nathan Kline Institute
74. University of Iowa College of Medicine
75. Cornell University
76. University of South Floriday: USF Health Byrd Alzheimer’s Institute
