# Supplementary material for: Tau deposition patterns are associated with functional connectivity in primary tauopathies
Source: Nat Commun. 2022 Mar 15;13:1362. doi: 10.1038/s41467-022-28896-3 (PMC8924216; doi:10.1038/s41467-022-28896-3)
Supplement: Supplementary file 3 — Reporting summary [file 41467_2022_28896_MOESM3_ESM.pdf]

nature portfolio

Corresponding author(s): Dr. Nicolai Franzmeier

Last updated by author(s): Feb 1, 2022

Reporting Summary

Nature Portfolio wishes to improve the reproducibility of the work that we publish. This form provides structure for consistency and transparency in reporting. For further information on Nature Portfolio policies, see our Editorial Policies and the Editorial Policy Checklist.

Statistics

For all statistical analyses, confirm that the following items are present in the figure legend, table legend, main text, or Methods section.

n/a	Confirmed	
☐	☒	The exact sample size (n) for each experimental group/condition, given as a discrete number and unit of measurement
☐	☒	A statement on whether measurements were taken from distinct samples or whether the same sample was measured repeatedly
☐	☒	The statistical test(s) used AND whether they are one- or two-sided<br>*Only common tests should be described solely by name; describe more complex techniques in the Methods section.*
☐	☒	A description of all covariates tested
☐	☒	A description of any assumptions or corrections, such as tests of normality and adjustment for multiple comparisons
☐	☒	A full description of the statistical parameters including central tendency (e.g. means) or other basic estimates (e.g. regression coefficient) AND variation (e.g. standard deviation) or associated estimates of uncertainty (e.g. confidence intervals)
☐	☒	For null hypothesis testing, the test statistic (e.g. F, t, r) with confidence intervals, effect sizes, degrees of freedom and P value noted<br>*Give P values as exact values whenever suitable.*
☒	☐	For Bayesian analysis, information on the choice of priors and Markov chain Monte Carlo settings
☒	☐	For hierarchical and complex designs, identification of the appropriate level for tests and full reporting of outcomes
☒	☐	Estimates of effect sizes (e.g. Cohen's d, Pearson's r), indicating how they were calculated

Our web collection on statistics for biologists contains articles on many of the points above.

Software and code

Policy information about availability of computer code

Data collection	no software was used
Data analysis	All analyses were computed using R statistical software 4.0.4

For manuscripts utilizing custom algorithms or software that are central to the research but not yet described in published literature, software must be made available to editors and reviewers. We strongly encourage code deposition in a community repository (e.g. GitHub). See the Nature Portfolio guidelines for submitting code & software for further information.

Data

Policy information about availability of data

All manuscripts must include a data availability statement. This statement should provide the following information, where applicable:

- Accession codes, unique identifiers, or web links for publicly available datasets
- A description of any restrictions on data availability
- For clinical datasets or third party data, please ensure that the statement adheres to our policy

The fMRI data that used in this study were obtained from the Alzheimer's disease Neuroimaging Initiative (ADNI) and are available from the ADNI database (adni.loni.usc.edu) upon registration and compliance with the data usage agreement. A list of ADNI RIDs that have been used for the current study can be obtained from the corresponding authors upon proof that data to ADNI has been granted. ADNI neuroimaging data (unprocessed or processed) are available from the corresponding authors upon request and upon proof of approved access to the ADNI database. Neuroimaging data (i.e. unprocessed or processed PET & MRI images) of PSP and CBS patients, as well as spreadsheets with post-mortem and autoradiography data from PSP and CBS patients are available under restricted access from the corresponding author upon request and approval of a dedicated data usage agreement between institutions exchanging data. Data sharing of all data used in the current study is restricted since ethics approvals for PET and Post-mortem studies or ADNI terms of use do not allow unrestricted and open-source

sharing of patient data with third-parties. Source data are provided with this paper. Source data are provided for all figures and supplementary figures showing individual datapoints and can be found in the supplementary.

Field-specific reporting

Please select the one below that is the best fit for your research. If you are not sure, read the appropriate sections before making your selection.

[x] Life sciences [ ] Behavioural & social sciences [ ] Ecological, evolutionary & environmental sciences

For a reference copy of the document with all sections, see nature.com/documents/nr-reporting-summary-flat.pdf

Life sciences study design

All studies must disclose on these points even when the disclosure is negative.

Sample size	PET sample, n = 61; Post-mortem sample, Munich, n = 97; Post-mortem sample UPENN, n = 96; ADNI fMRI sample, n = 69; samples were derived from previous studies, no sample size calculation was performed
Data exclusions	No data was excluded from the analyses
Replication	Findings were validated across PET-imaging and post-mortem data, with replication in two independent post-mortem datasets
Randomization	No randomization was performed, since no treatment/intervention is investigated
Blinding	No blinding was performed, since no treatment/intervention is investigated

Reporting for specific materials, systems and methods

We require information from authors about some types of materials, experimental systems and methods used in many studies. Here, indicate whether each material, system or method listed is relevant to your study. If you are not sure if a list item applies to your research, read the appropriate section before selecting a response.

Materials & experimental systems

n/a	Involved in the study	
[ ]	[x]	Antibodies
[x]	[ ]	Eukaryotic cell lines
[x]	[ ]	Palaeontology and archaeology
[x]	[ ]	Animals and other organisms
[ ]	[x]	Human research participants
[x]	[ ]	Clinical data
[x]	[ ]	Dual use research of concern

Methods

n/a	Involved in the study	
[x]	[ ]	ChIP-seq
[x]	[ ]	Flow cytometry
[ ]	[x]	MRI-based neuroimaging

Antibodies

Antibodies used	AT8 (Ser202/Thr2015, 1:200, Invitrogen/Thermofischer, MN1020, Carlsbad, USA); PHF-1 (Ser396/Ser404, 1:2000; Gift of Peter Davies)
Validation	Antibodies are established for post-mortem assessments of fibrillary tau pathology (e.g. Kovacs et al., Acta Neurpath, 2020). No specific validation steps have been undertaken for this study.

Human research participants

Policy information about studies involving human research participants

Population characteristics	For the imaging study, we included 61 subjects recruited at four sites (Munich & Leipzig, Germany; Melbourne, Australia; New Haven, United States), including 15 cognitively normal individuals (i.e. without evidence of cognitive decline, any motor symptoms or cerebral tau pathology, mean age 63±9, 9 women, 6 men), 24 patients with clinical diagnosis of possible or probable cortico-basal syndrome (CBS, mean age 68±8, 14 women, 10 men) and 22 patients with clinical diagnosis of PSP-RS (mean age 72±6, 10 women, 18 men). CBS diagnosis was made according to the revised Armstrong Criteria of probable CBS or the Movement Disorders Society criteria of possible PSP with predominant CBS.33 PSP-RS was diagnosed following state-of-the-art diagnostic criteria.
Recruitment	All patient data derive from a PSP cohort recruited in Munich and Leipzig and a CBS cohort recruited in Munich. To replicate tau-PET vs. connectivity associations using post-mortem assessments of tau pathology, we included histopathological tau data from two independent samples, including n=97 PSP subjects recruited, sampled and examined at several different sites across Europe with centralized final analysis at the Department of Neuropathology, LMU, in Munich and n=96 PSP subjects from the University of Pennsylvania. An in-depth description of data selection and data acquisition has been published previously (Kovacs et al., Acta Neuropath, 2020)
Ethics oversight	The 18-F-PI2620 PET imaging protocol was approved by local ethics committee of the LMU Munich. Written informed consent was obtained from all participants. The full study protocol including all samples and all PET data analyses were approved by the local ethics committee (LMU Munich, application numbers 17-569 and 19-022) and the German radiation protection (BfS-application: Z 5 - 22464/2017-047-K-G) authorities. The study was carried out according to the principles of the Helsinki Declaration. The post-mortem examinations of brain tissue in PSP patients were approved by the ethics committee of the medical faculty of the university of Marburg, Germany.<br>All work complied with ethical regulations for work with human participants.

Note that full information on the approval of the study protocol must also be provided in the manuscript.

Magnetic resonance imaging

Experimental design

Design type	NA
Design specifications	NA
Behavioral performance measures	NA

Acquisition

Imaging type(s)	T1-weighted structural MRI, functional MRI
Field strength	3
Sequence & imaging parameters	T1, Echo Planar Imaging
Area of acquisition	brain

Diffusion MRI ☐ Used ☒ Not used

Preprocessing

Preprocessing software	ANTs, FSL
Normalization	ANTs
Normalization template	MNI
Noise and artifact removal	fMRI: To denoise the EPI images, we regressed out nuisance covariates (i.e. average white matter and cerebrospinal fluid signal and motion parameters estimated during motion correction), removed the linear trend and applied band-pass filtering with a 0.01-0.08Hz frequency band in EPI native space. To further minimize the impact of motion which may compromise FC assessment,70 we performed motion scrubbing, where we censored volumes that showed a frame-wise displacement of >1mm, as well as one prior and two subsequent volumes. In line with our previous work,71 only subjects for whom less than 30% of volumes had to be censored were included in the current study.
Volume censoring	To further minimize the impact of motion which may compromise FC assessment,70 we performed motion scrubbing, where we censored volumes that showed a frame-wise displacement of >1mm, as well as one prior and two subsequent volumes. In line with our previous work,71 only subjects for whom less than 30% of volumes had to be censored were included in the current study.

Statistical modeling & inference

Model type and settings	NA
Effect(s) tested	NA

Specify type of analysis: ☐ Whole brain ☐ ROI-based ☒ Both

Anatomical location(s): TIAN Atlas, Schaefer Atlas

Statistic type for inference (See Eklund et al. 2016)	Testing of group-differences in tau-PET SUVRs (ANOVA)
Correction	voxel threshold of p<0.005 with a cluster size of at least 100 spatially contiguous voxels

Models & analysis

n/a	Involved in the study
☐	☒ Functional and/or effective connectivity
☒	☐ Graph analysis
☒	☐ Multivariate modeling or predictive analysis

Functional and/or effective connectivity	Fisher-z transformed Pearson Correlation
